# Requirements for Eliciting a Spastic Response With Passive Joint Movements and the Influence of Velocity on Response Patterns: An Experimental Study of Velocity-Response Relationships in Mild Spasticity With Repeated-Measures Analysis

**DOI:** 10.3389/fneur.2022.854125

**Published:** 2022-03-30

**Authors:** Kenta Fujimura, Masahiko Mukaino, Shota Itoh, Haruna Miwa, Ryoka Itoh, Daisuke Narukawa, Hiroki Tanikawa, Yoshikiyo Kanada, Eiichi Saitoh, Yohei Otaka

**Affiliations:** ^1^Faculty of Rehabilitation, School of Health Sciences, Fujita Health University, Toyoake, Japan; ^2^Department of Rehabilitation Medicine I, School of Medicine, Fujita Health University, Toyoake, Japan; ^3^Department of Rehabilitation, Fujita Health University Hospital, Toyoake, Japan

**Keywords:** spasticity, resistance, elbow joint, velocity-dependent, clinical assessment

## Abstract

**Background:**

Spasticity is defined as a velocity-dependent increase in tonic stretch reflexes and is manually assessed in clinical practice. However, the best method for the clinical assessment of spasticity has not been objectively described. This study analyzed the clinical procedure to assess spasticity of the elbow joint using an electrogoniometer and investigated the appropriate velocity required to elicit a spastic response and the influence of velocity on the kinematic response pattern.

**Methods:**

This study included eight healthy individuals and 15 patients with spasticity who scored 1 or 1+ on the modified Ashworth Scale (MAS). Examiners were instructed to manually assess spasticity twice at two different velocities (slow and fast velocity conditions). During the assessment, velocity, deceleration value, and angle [described as the % range of motion (%ROM)] at the moment of resistance were measured using an electrogoniometer. Differences between the slow and fast conditions were evaluated. In addition, variations among the fast condition such as the responses against passive elbow extension at <200, 200–300, 300–400, 400°/s velocities were compared between the MAS 1+, MAS 1, and control groups.

**Results:**

Significant differences were observed in the angular deceleration value and %ROM in the fast velocity condition (417 ± 80°/s) between patients and healthy individuals, but there was no difference in the slow velocity condition (103 ± 29°/s). In addition, the deceleration values were significantly different between the MAS 1 and MAS 1+ groups in velocity conditions faster than 300°/s. In contrast, the value of %ROM plateaued when the velocity was faster than 200°/s.

**Conclusion:**

The velocity of the passive motion had a significant effect on the response pattern of the elbow joint. The velocity-response pattern differed between deceleration and the angle at which the catch occurred; the value of deceleration value for passive motion was highly dependent on the velocity, while the %ROM was relatively stable above a certain velocity threshold. These results provide clues for accurate assessment of spasticity in clinical practice.

## Introduction

Spasticity is a positive sign of upper motor neuron syndrome caused by cerebrovascular or spinal cord injury and is defined as “a motor disorder characterized by a velocity-dependent increase in tonic stretch reflexes (“muscle tone”) with exaggerated tendon jerks, resulting from hyperexcitability of the stretch reflex, as one component of the upper motor neuron syndrome” (1). In the clinical setting, the severity of spasticity is usually assessed manually using the response evoked when the spastic muscle is rapidly stretched by passive movement, which causes rapid muscle activity that generates resistance to movement (2).

Manually evoked spastic muscle responses are evaluated using clinical scales. The Ashworth Scale (AS) (3), modified Ashworth scale (MAS) (4), and modified Tardieu scale (MTS) are the most commonly used clinical scales for the assessment of spasticity (5). The angle at which resistance occurs, presence of spastic dystonia, and the strength of resistance related to this passive stretching are the chief indicators of the severity of spasticity in these clinical scales. Although these clinical scales are widely used in clinical settings, there is some debate regarding the reliability of these scales. Numerous studies have provided conflicting evidence on the reliability of the AS, MAS, and MTS. For example, several reports have demonstrated good intra- and inter-rater reliability for AS (6) and MAS (4, 7), while others have demonstrated poor reliability (8–10). One study showed that the inter-rater reliability of MTS was higher than that of MAS (11); conversely, numerous studies have questioned the inter-rater reliability of MTS for clinical evaluation (12, 13).

One possible reason for the recurrent debate on the reliability of these clinical scales may be related to ambiguity in how the spasticity-testing technique is defined (14, 15). For example, in evaluating MTS, the velocity is roughly categorized into three levels: (1) “as slow as possible,” (2) “gravitational velocity,” and (3) “as fast as possible.” Considering the velocity-dependent nature of spasticity, the angle at which resistance occurs and the strength of resistance may change depending on the passive movement velocity; thus, the testing maneuver should be more rigorously defined.

There is a more concrete description of the practical technique used in the MAS, described by Bohannon et al. (4) as to “extend the patient's elbow from a position of maximal possible flexion to maximal possible extension over a duration of about 1 s.” However, the assessed velocity can still vary considerably, as the range of motion differs from one patient to another. In addition, it is not clear whether the velocity with which an examiner moves the patients' elbow through the full range of motion in a second is the best technique to elicit and distinguish spastic response. This is especially relevant in patients with mild spasticity. For example, patients with MAS2 or higher show resistance throughout the range of motion which is a typical sign during clinical evaluation. However, the dynamic spastic response which the raters rely on in evaluating MAS1 and MAS1+ can easily vary with velocity (16, 17), making it difficult at times to detect symptoms and to distinguish its severity. Therefore, an accurate understanding of the velocity-response relationship would be essential.

This study aimed to clarify the influence of velocity on the kinematic response patterns in poststroke spasticity, especially in patients with mild spasticity. We sought to do this by using an electronic goniometer to measure the influence of velocity on the resistance-response pattern against passive movement.

## Materials and Methods

### Participants

Participants were recruited from among those patients who underwent rehabilitation at Fujita Health University in Toyoake, Japan, between February and April 2020 and from the local community. The study population comprised a total of 23 participants, of whom 15 were patients with stroke with hemiplegia and spasticity (spasticity group) and eight were healthy individuals without any history of central nervous system or musculoskeletal diseases (control group).

Inclusion criteria for the spasticity group were as follows: (1) existence of spasticity in the elbow flexor muscle group and (2) ischemic or hemorrhagic stroke. Exclusion criteria included: (1) presence of a manually detected increase in muscle tone during slow stretching; (2) motor, cognitive, visual, or hearing dysfunction that hindered spasticity measurements; and (3) limitation in the passive range of motion (ROM) in the paretic upper limb. Participant details are presented in Table 1. This study was approved by our institutional review committee, and written informed consent was obtained from all participants.

**Table 1:** Demographic variables.

	**Spasticity group**	**Control group**
Number	15	8
Male: Female	10:5	5:3
Age (mean ± SD)	51 ± 15	26 ± 3
Height [cm] (mean ± SD)	163.9 ± 10.3	165.5 ± 7.2
Weight [kg] (mean ± SD)	63.1 ± 14.5	58.4 ± 11.1
Paralyzed side	Right:Left = 4:11	–
Time after onset [days] (mean ± SD)	926 ± 1,433	–
Severity of spasticity (MAS 1/1+)	7/8	–

*SD, standard deviation; MAS, modified Ashworth scale*.

### Procedure

For all assessments, the participant was placed in a sitting position with a backrest, and an electronic goniometer (Biometrics, Ladysmith, USA; sampling frequency 1,000 Hz) was attached to the elbow joint. Two of six randomly selected physiotherapists and occupational therapists (mean 4.3 years of clinical experience) performed all examinations. The examiner held the participant's elbow joint in maximum flexion for 2 s and then moved it to maximum extension and measured the elbow joint angle during this period. Examiners were instructed to test joint mobility under two different velocities: (1) a slow-speed condition to examine the ROM and (2) a fast-speed condition to evaluate spasticity. Two examiners performed the test twice under each speed condition for each participant. Thus, we obtained four sets of time-angle data at low-speed conditions and four datasets at high-speed conditions for each study participant. The minimum speed among the four datasets of the low-velocity condition was set as the representative value for the slow condition (henceforth referred to as Slow), and the maximum speed among the four datasets for the high-speed condition was set as the representative data for the fast condition (henceforth referred to as Fast) for each participant.

### Data Analysis

**Figure 1:**
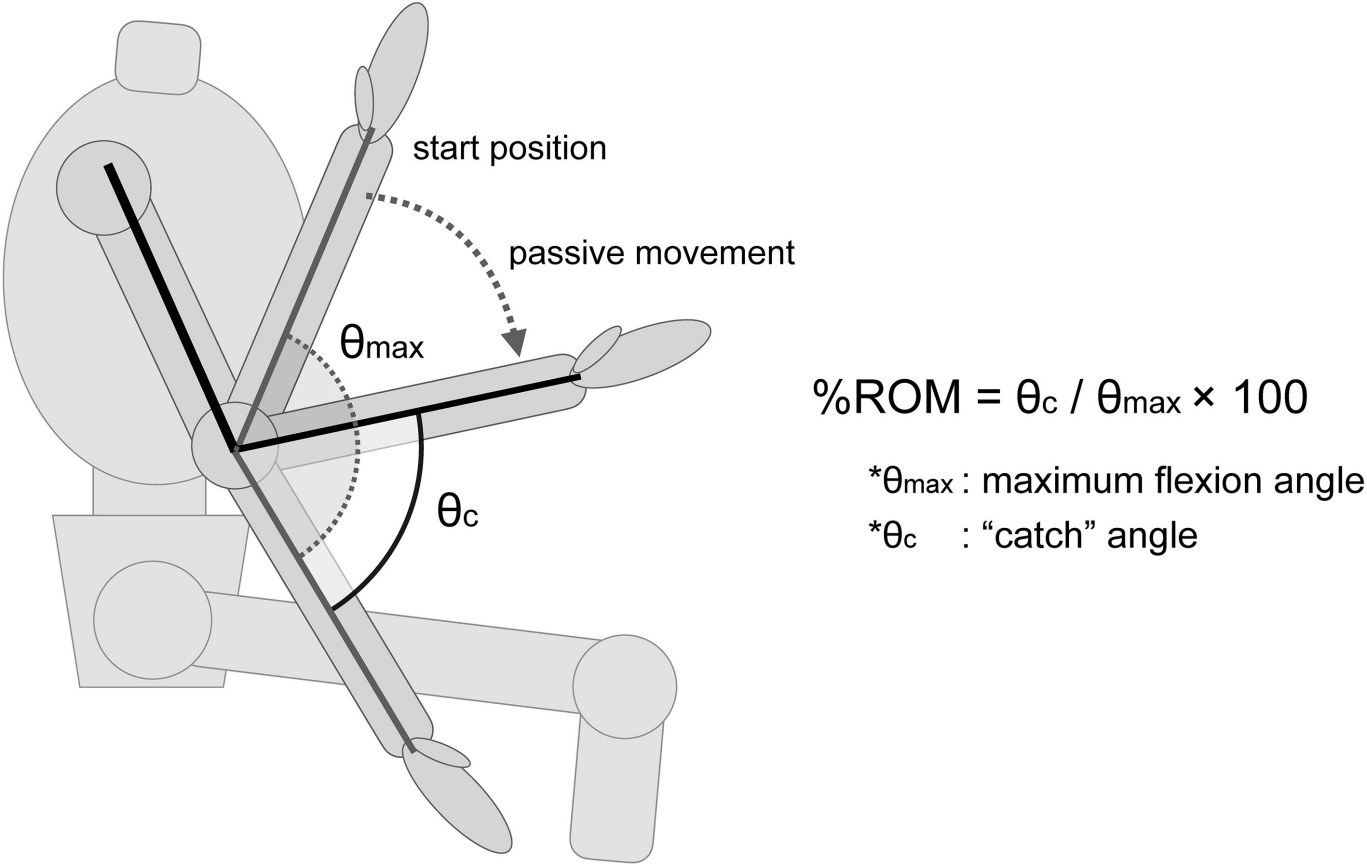
Description of the %ROM. The %ROM was calculated as the ratio of the angle at which the “catch” of the spasticity occurs. θmax, maximum flexion angle of the elbow joint. θc, the angle at which the “catch” of the spasticity muscle occurs.

We calculated the velocity, deceleration value, and %ROM during passive movement using the gathered time-angle data. Each index value was defined as follows:

- “Catch” angle (θ_c_): the elbow joint angle that indicates the occurrence of the “catch.” Although the “catch” is typically considered the stop of motion where the velocity goes to zero, such a complete cessation of movement may not always occur, depending on the velocity and the degree of spasticity. To detect a wider range of dynamic spasticity responses, the angle of “catch” in this study was defined as the angle from the start of joint motion to the point where deceleration by braking begins. To eliminate the influence of small fluctuations in acceleration value, we defined the zero value of acceleration by a range of ±3 standard deviations (SD); the start of the movement and the start of the deceleration were defined as the points at which deceleration value exceeded the mean ± 3 SD of the acceleration at rest and the point when it returned to within the mean ± 3 SD, respectively.
- Maximum velocity (°/sec): maximum value of velocity
- Maximum deceleration value at the “catch” (°/sec^2^): maximum absolute deceleration value
- %ROM (%): “catch” angle (θ_c_) / maximum flexion angle (θ_max_) × 100 (Figure 1).

**Figure 2:**
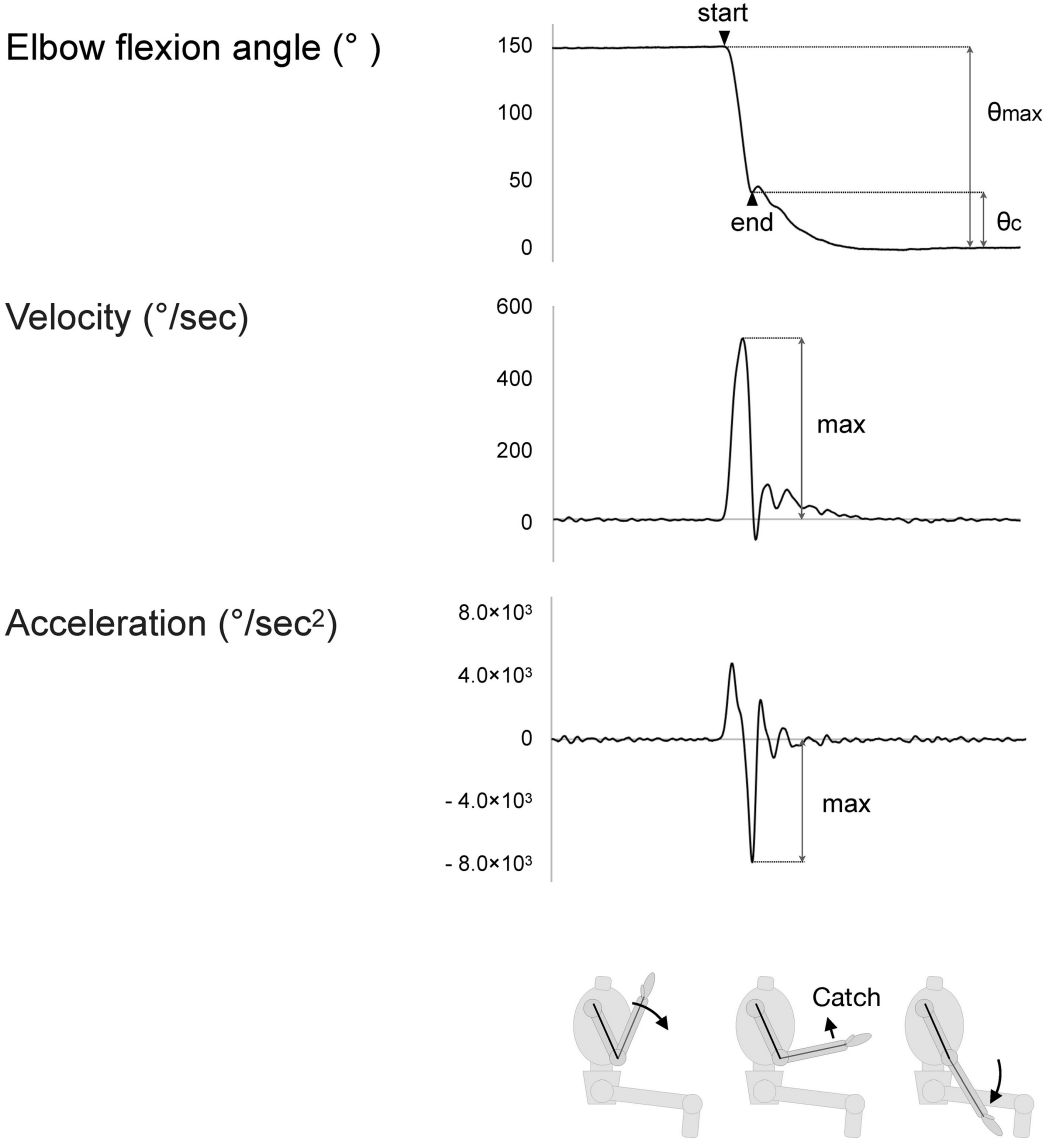
The maximum values of indices. Velocity rises as passive movement in the elbow joint begins. Acceleration similarly rises but falls with deceleration of the motion. The value of deceleration is considered to reflect the strength of the “catch” of the spastic muscle.

Examples of the index values are shown in Figure 2.

The deceleration value and %ROM values at the Slow or Fast conditions were compared between the spasticity and control groups. Then, the 15 patients in the spasticity group were classified into two subgroups according to the severity of their measured spasticity (MAS1 and 1+), and the deceleration value and % ROM during passive movement of the MAS 1, MAS 1+, and control groups were compared. To investigate the relationships between spastic response and velocity, the measurement data of the deceleration value and % ROM (144 datapoints total from 23 participants with eight datasets for each) were classified into four velocity conditions (<200, 200–300, 300–400, 400 < °/s) according to the velocity actually applied and compared within each condition. If there were two or more datapoints for the same participant within the same velocity condition, we used the average of these datapoints for our calculations. We used two-way repeated measures analysis of variance (ANOVA) for statistical analysis, and *post-hoc* multiple comparisons were performed using Tukey's HSD test, when significant. The significance level was set at 5%. The average data was shown with the SD.

## Results

**Figure 3:**
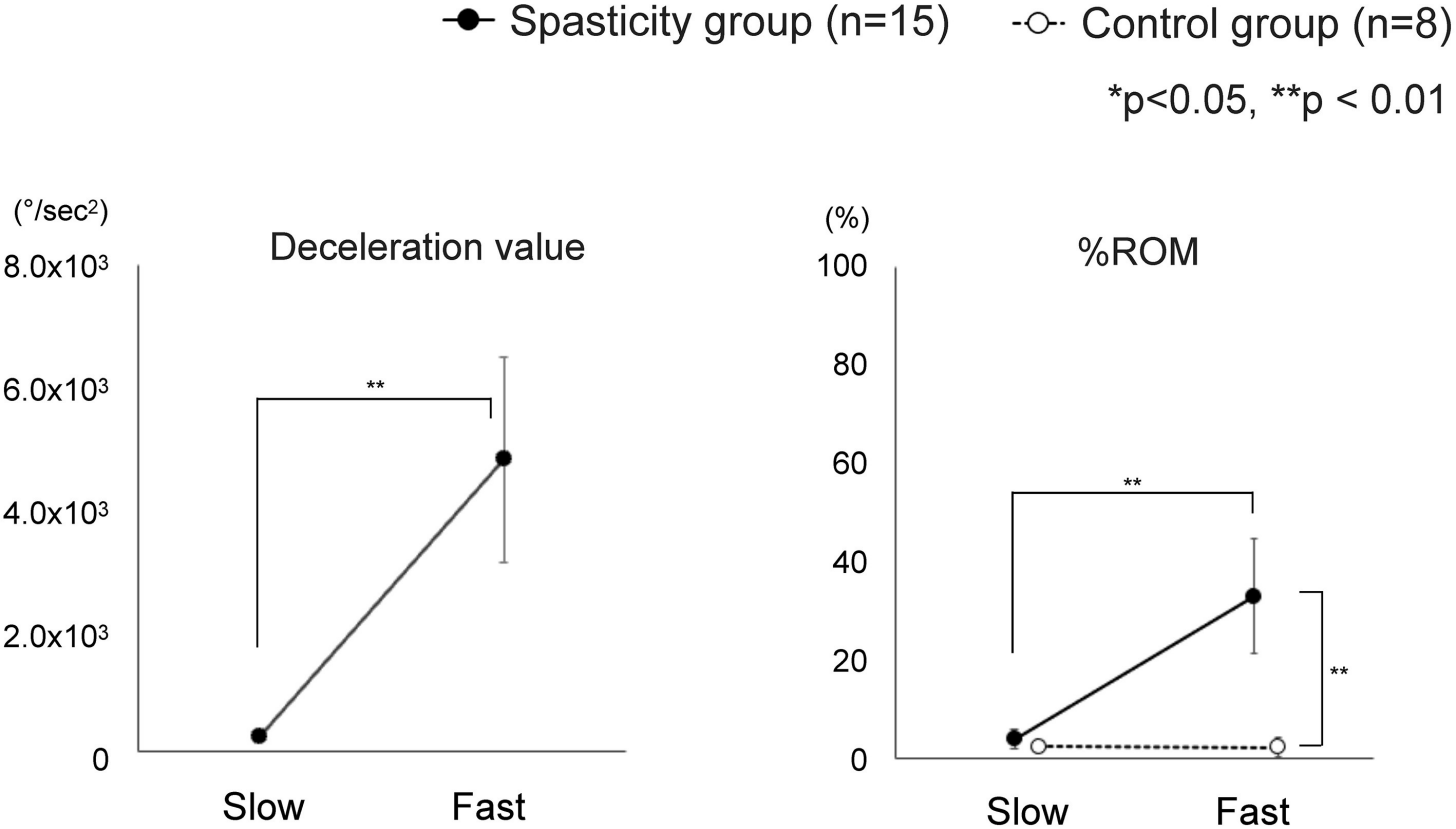
Change in index values between Slow and Fast conditions. The spasticity group showed significant differences in all index values between Slow and Fast. Comparison between the spasticity group and the control group showed significant difference in the Fast condition for %ROM. ^*^Slow: The minimum speed among the four datasets of the low-speed condition. ^*^Fast: The maximum speed among the four datasets of the high-speed condition.

In total, 23 individuals including 15 patients suffering from stroke with hemiplegia and spasticity (seven MAS1 and eight MAS1+ patients) and eight healthy individuals participated in this study. All the participants underwent complete measurements. The average maximum velocities in the Slow and Fast conditions were 103 ± 29 and 417 ± 80°/s, respectively (mean ± SD). No significant differences were observed between the spasticity and control groups (Table 2). The maximum deceleration and %ROM at Slow and Fast are shown in Figure 3. The deceleration value in patients with spasticity was significantly greater in Fast condition than that in Slow (4,814 ± 1,689 vs. 260 ± 57, *P* < 0.001). Repeated-measures ANOVA of %ROM with group factor (patient or control) and velocity factor (Slow or Fast) showed significant effects for group factor (*F* = 11.2, df = 1, *P* < 0.001), velocity factor (*F* = 15.5, df = 1, *P* < 0.001), and group × velocity interaction (*F* = 10.1, df = 1, *P* = 0.005). The *post-hoc* multiple comparison test indicated that the %ROM was significantly greater in the Fast condition compared to the Slow condition in the spasticity group (33 ± 12 vs. 4 ± 2, *P* < 0.001). In the Slow condition, there was no significant difference in %ROM between the spasticity group and control group. However, in the Fast condition, %ROM was significantly higher in the spasticity group compared to the control group (33 ± 12 vs. 12 ± 2, *P* < 0.001; Figure 3).

**Table 2:** Angular velocity at Slow and Fast conditions.

	***n***	**Slow (degree)**			**Fast (degree)**			***P-*value (Slow vs. Fast)**
Spasticity group	15	107 ± 30	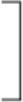	*P* = 0.9844	411 ± 90	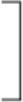	*P* = 0.9220	<0.0001
Control group	8	97 ± 28			428 ± 60			<0.0001

*mean ± SD; SD, standard deviation*.

**Figure 4:**
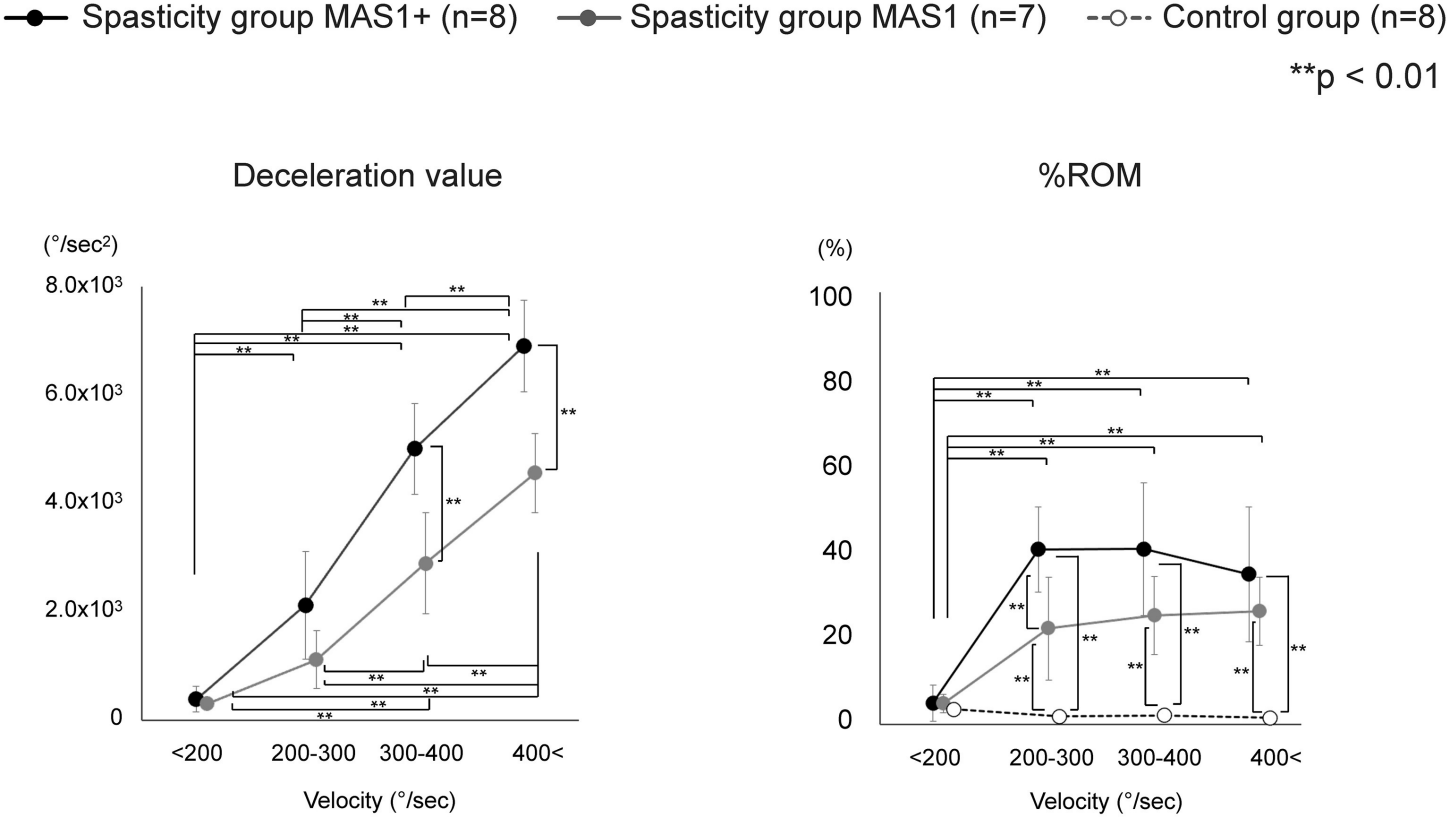
Variation of each index value with change in velocity. Deceleration value increased with increasing velocity, and there was also a significant difference between MAS 1+ and MAS 1 above 300°/s conditions. Unlike deceleration value, the value of the %ROM difference between MAS 1+ and MAS 1 occurred only in 200–300°/s conditions and did not increase with an increase in the velocity. The %ROM of control was significantly lower than that of the spasticity groups.

As there was marked variation in the measured velocity applied by the examiners, we analyzed the data using four different velocity groups (<200, 200–300, 300–400, and 400 < °/s; Table 3). The values of deceleration and %ROM in the four velocity conditions are depicted in Figure 4. Repeated-measures ANOVA of deceleration value with MAS factor (MAS1 and 1+) and velocity factor (<200, 200–300, 300–400, or 400 < ) showed significant effects for MAS factor (*F* = 23.1, df = 1, *P* < 0.001), velocity factor (*F* = 163.8, df = 3, *P* < 0.001), and group × velocity interaction (*F* = 7.7, df = 3, *P* < 0.001). Repeated-measures ANOVA of %ROM with MAS factor (MAS1, 1+ or control) and velocity factor (<200, 200–300, 300–400, 400 < ) showed significant effects for MAS factor (*F* = 49.4, df = 2, *P* < 0.001), velocity factor (*F* = 41.7, df = 3, *P* < 0.001), and group × velocity interaction (*F* = 16.3, df = 6, *P* < 0.001). The *post-hoc* tests indicated a significant difference in the deceleration value between all velocity conditions in the spasticity group with MAS 1+ (<200 vs. 200–300 vs. 300–400 vs. 400 < °/s: 390 ± 232 vs. 2,124 ± 990 vs. 5,011 ± 836 vs. 6,906 ± 842, all *P* < 0.001). In the spasticity group with MAS 1, there were significant differences between all the velocity conditions except for velocity conditions <200 vs. 200–300°/s (299 ± 53 vs. 1,121 ± 535 vs. 2,894 ± 932 vs. 4,562 ± 735, all *P* < 0.001). We observed significant differences between the spasticity group with MAS 1+ vs. the one with MAS 1, MAS 1 vs. the control group in 300–400 and 400 < °/s conditions (MAS 1+ vs. MAS 1: both *P* < 0.001).

**Table 3:** Average velocities in four velocity conditions.

**Velocity conditions**	** <200**			**200-300**			**300-400**			**400 < **	
Spasticity group (MAS 1+)	105 ± 36	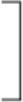 ns	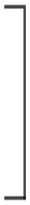	254 ± 35	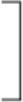 ns	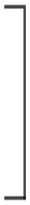	365 ± 15	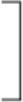 ns	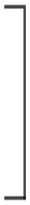	468 ± 36	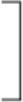 ns	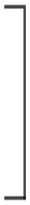
Spasticity group	145 ± 21		ns	250 ± 26		ns	346 ± 13		ns	489 ± 62		ns
(MAS 1)		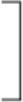 ns			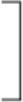 ns			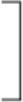 ns			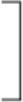 ns	
Control group	110 ± 26			238 ± 29			348 ± 21			471 ± 32		

*mean ± SD; SD, standard deviation; ns, no significant*.

In %ROM, there were significant differences between <200°/s and the other three conditions in the MAS 1+ and 1 spasticity groups (MAS 1+: all *p* < 0.001, MAS 1: <200 vs. 200–300, <200 vs. 300–400, <200 vs. 400 < °/s: *P* = 0.002, *P* < 0.001, *P* < 0.001, respectively). Unlike deceleration value, the value of %ROM did not increase with the velocity increase, and there was no significant difference in the %ROM between the 200–300, 300–400, and 400 < °/s conditions. The control group showed no difference between all velocity conditions. Comparison of %ROM between the groups in each velocity condition showed significant differences between the spasticity subgroups with MAS 1+ and MAS 1 vs. the control group at 200–300, 300–400, and 400 < °/s (MAS 1+ vs. control group: all *P* < 0.001, MAS 1 vs. control group: *P* = 0.002, *P* < 0.001, *P* < 0.001, respectively). Moreover, there were significant differences between the spasticity group with MAS 1+ vs. MAS 1 in only the 200–300°/s conditions (*P* = 0.002). There were no differences between the three groups at <200°/s conditions.

## Discussion

In the present study, we clarified the relationship between passive motion velocity and reaction patterns in spastic elbow joints with mild spasticity. Specifically, deceleration value, which indicates the strength of the braking response, and %ROM, which indicates the angle at which the response occurs, differed in their responses to motion velocity. The value of deceleration for motion climbed with increasing speed. This surge was greater in patients (spasticity group) than that in the healthy participants (control group), and there were significant differences between the patients and healthy participants in the Fast condition, while there were no differences in the Slow condition. Moreover, significant differences in deceleration value between the MAS 1 and MAS 1+ groups were observed in the conditions where the speed was faster than 300°/s. Movement velocity also affected the %ROM at which the catch occurs. However, the response patterns differed from that of the deceleration value; the %ROM was higher in the Fast condition than that in the Slow condition, but no significant difference was observed between the conditions with velocity faster than 200°/s. A significant difference between MAS 1 and MAS1+ in %ROM was observed at the velocity of 200–300°/s.

The deceleration value as resistance to the movement represents braking against the movement due to the spastic response, reflecting the magnitude of resistance force. Previous studies have shown that electromyographic responses and resistance increase with increasing velocity in a passive extension of spastic joints, which is consistent with the results of the present (18–22). In the present study, differences between groups (MAS 1 and MAS 1+) were more evident than in previous studies on the mechanical measurement of elbow spasticity (21, 22). This may be attributed to the adoption of a faster velocity than in the previous studies, based on the substantial velocity dependence of the braking response shown in the present study. Therefore, to distinguish the degree of spasticity by the strength of the response, tests should be performed at very high speeds; based on the present study results, the test speed should be more than 300°/s.

The difference in %ROM between patients and healthy subjects was evident for passive exercise in the Fast condition; however, not apparent in the Slow condition. In addition, no significant differences in %ROM between the velocity conditions of 200–300, 300–400, and 400 and above were observed in either of the subgroups (MAS1+, MAS1, and control). These results show that there is a ceiling effect for the velocity that induces a catch. Calota et al. reported that there is a correlation between velocity and the angle at which the spastic response appears, and that the tonic stretch reflex threshold (TSRT), the intercept calculated from the linear regression, is a reliable indicator of spasticity in patients with moderate to severe spasticity (17). Similarly, the present study confirmed the velocity-response angle relationship; however, a ceiling effect was observed at fast velocities. In their report, Calota et al. indicated that TSRT may not be a reliable indicator for mild spasticity. The ceiling effect observed in the present study with patients with mild spasticity may relate to this poor fit of linear regression and low reliability of TSRT in mild spasticity.

On the other hand, the velocity-angle relationships shown in this study may be more complex in more severe cases that experience spastic dystonia, which is the most frequently observed symptom in patients with poststroke hypertonia (23). Spastic dystonia is considered to be a separate symptom from spasticity; while spasticity is typically characterized by velocity-dependent hypertonia and tendon-jerk hyperreflexia, spastic dystonia refers to the relative inability to relax muscles (24, 25). Since this study included only patients without manually detected static increase in muscle tone, the present results are expected to mostly reflect the increase in dynamic tonic reflex elicited during passive movement that is derived from the spasticity. However, in patients with severe spastic dystonia, which is also a velocity-dependent symptom with different clinical features (25), the clinical manifestation of poststroke hypertonia can be different. For the holistic understanding of relationships between the movement velocity and kinematic response, further studies should be conducted to investigate the impact of spastic dystonia on the velocity-response relationship.

## Limitations

First, the movement velocities in each test varied because the participants of this study underwent manual testing rather than automated testing that is inherently objective. We did not define the condition in advance since we endeavored to obtain clues from the clinical testing procedure. Although this method replicated the actual evaluation of spasticity in clinical practice, it might be insufficient to examine the effect of velocity in detail. Further studies that examine resistance response at a uniform velocity will promote our understanding of the relationship between movement velocity and spastic response.

Second, the study population was limited to patients with mild spasticity, rated as MAS 1 or MAS 1+. We opted to enroll in our study patients with mild spasticity because patients with severe spasticity usually present with increased stiffness in soft tissue and enhanced neural response, either of which would require a more complex analytical procedure than that examined here, as mentioned above. Future studies with patients with more severe spasticity are needed to evaluate this point.

Lastly, electromyography was not conducted in this study. We focused on the kinematic response that was directly related to the clinician's clinical maneuver. However, to investigate the mechanism behind this velocity-response relationship further, especially on the contribution of spastic dystonia, an electromyography study is necessary.

## Clinical Implications

While spastic dystonia in patients with MAS2 and above is a relatively clear-cut symptom that can be observed at rest, the detection of a velocity-dependent response such as catch in mild spasticity including MAS1 and MAS1+ may vary greatly depending on the examiner's technique. The present results showed a possible great variation in catch detection in clinical maneuvers; the strength of response and the catch angle varied extensively with velocity. Given the large variation in velocity in measurement procedures seen in the present study, it is possible that reliability problems with clinical scales such as MAS and MTS may be linked to the variations in procedures and therefore a strict definition of measurement conditions may improve the reliability of clinical scales.

In particular, the strong dependence of the extent of braking response on the movement velocity seen in this study may indicate that the clinicians should not be influenced by the strength of braking response in manually evaluating the severity of spasticity where the speed of movement varies. For example, the strength of braking response in MAS1 patients may be greater than that in MAS1+ patients if the velocity was faster by 100–200°/s. However, the apparent differences in deceleration values between the groups in high-velocity conditions (faster than 300°/s) imply that the measurement of deceleration value can be useful when the measurement is performed in strictly fixed velocity conditions, which may be achieved by mechanical measurement.

In %ROM, the differences between MAS 1+, MAS 1, and control could be detected in the velocity of 200–300°/s. Interestingly, there was no significant difference in %ROM between the conditions with a velocity of 200–300, 300–400, and >400°/s. Therefore, when the velocity is sufficiently high, the %ROM should be considered a stable indicator of spasticity in manual testing, where the velocity can vary among different testers. This may also support the reliability of existing clinical scales that depends on the angle of the catch in their assessment. However, the definition of the velocity used in the testing procedure may have to be updated. Previously, the MAS testing procedure was defined as being done by “passively stretching the muscles through their available range of motion over a period of about 1 s” (4). This equals ~140–145°/s in the elbow joint if there is no limitation in range of motion (26). However, the results of this study indicate that this velocity needs to be faster in order to reliably detect a catch; to move the joint at a velocity of 200–300°/s, the time to move the elbow joint through the full range of motion is suggested to be 0.5–0.7 s. A detailed definition of the testing maneuver based on these findings may improve the reliability of MAS. On the other hand, in MTS, the angle measurement for catch detection is defined only as “as fast as possible.” This ambiguity might lessen the reliability of the MTS as indicated previously (12). However, by setting clear specifications, the scale may become more reliable. In addition, the use of an electronic goniometer, such as the one used in this study, may improve the measurement accuracy and further contribute to the reliability of MTS. With higher confidence in its reliability, MTS, which includes the angle at which catches and clonus occurs as a quantitative parameter, should be of increasing value in clinical practice as a sensitive measure of changes in spasticity.

In summary, kinematic and dynamic analysis of passive elbow movements at varying speeds in patients diagnosed with mild spasticity confirmed the velocity dependence of hypertonia, which include spasticity and possible spastic dystonia, and provided more details of symptoms than clinical scales at the individual patient level. A novel result of the present study is that kinematic measures, especially angles, are good markers of hypertonia at velocity of at least 200°/s. This finding suggests that the velocity of passive movement, when subjecting patients to MAS, should be updated; in addition, this velocity requirement may also be better considered in using MTS to improve its reliability.

## Conclusions

Here we examined the resistance response of spastic muscles to manual passive movement in clinical practice based on indices such as deceleration value and %ROM. Our results suggest that a catch against the passive movement is steadily observed in velocities faster than 200°/s and that the values of maximum deceleration in resisting the passive extension of the elbow joint are highly velocity-dependent. Conversely, the angle at which the catch against the passive joint movement occurred did not change significantly with the velocity of the movement in velocities faster than 200°/s. Therefore, the joint angle at which the resistance reaction occurs can be a stable indicator of the severity of the spasticity. Future studies that include patients with increased static muscle stiffness are warranted to further understand the velocity-dependent response to passive movements in patients with spasticity.

## Data Availability Statement

The raw data supporting the conclusions of this article will be made available by the authors, without undue reservation.

## Ethics Statement

The studies involving human participants were reviewed and approved by Fujita Health University Ethics Committee. The patients/participants provided their written informed consent to participate in this study.

## Author Contributions

KF, MM, SI, HM, RI, DN, and HT designed and conducted the study, participated in data collection, and conducted data analysis. KF and MM prepared the manuscript draft with important intellectual input from YK, ES, and YO. All authors reviewed and approved the final manuscript.

## Conflict of Interest

The authors declare that the research was conducted in the absence of any commercial or financial relationships that could be construed as a potential conflict of interest.

## Publisher's Note

All claims expressed in this article are solely those of the authors and do not necessarily represent those of their affiliated organizations, or those of the publisher, the editors and the reviewers. Any product that may be evaluated in this article, or claim that may be made by its manufacturer, is not guaranteed or endorsed by the publisher.
